# Effect of Butadiene Rubber Crystallization on Low-Temperature Properties of Butadiene/Silicone Rubber Blends with Potential for Mars Applications

**DOI:** 10.3390/ma17194857

**Published:** 2024-10-02

**Authors:** Norbert Nizel, Dariusz M. Bieliński, Andrzej Pawlak, Magdalena Maciejewska, Jakub Wręczycki, Marcin Masłowski, Rafał Anyszka

**Affiliations:** 1 Institute of Polymer and Dye Technology, Faculty of Chemistry, Lodz University of Technology, Stefanowskiego 16, 90-537 Lodz, Poland; dariusz.bielinski@p.lodz.pl (D.M.B.); magdalena.maciejewska@p.lodz.pl (M.M.); jakub.wreczycki@p.lodz.pl (J.W.); marcin.maslowski@p.lodz.pl (M.M.); 2 Centre of Molecular and Macromolecular Studies, Polish Academy of Sciences, Sienkiewicza 112, 90-363 Lodz, Poland; andrzej.pawlak@cbmm.lodz.pl

**Keywords:** butadiene rubber, silicone rubber, crystallization, low-temperature properties, Mars, polymer blends, thermal shrinkage

## Abstract

This study explores the impact of butadiene rubber (BR) crystallization on the low-temperature properties of butadiene/silicone (VMQ) rubber blends (BR/VMQ) designed for Martian applications. Two types of BR, semi-crystalline high-cis Buna CB24 and amorphous Buna CB550, were blended with VMQ, and their mechanical and thermal properties were evaluated. Kinetics of vulcanization, static mechanical properties, dynamical mechanical analysis, thermal shrinkage, and differential scanning calorimetry were utilized. The research demonstrates that the semi-crystalline BR improves mechanical properties but induces greater shrinkage at low temperatures. Conversely, using amorphous BR provided more consistent mechanical properties across the Martian temperature range and reduced material shrink by 6.71% for samples with carbon black, by 8.36% for samples with silica, and by 11.63% for unfilled samples. Future research will be required to evaluate the impact of volume change on the sealing properties of the BR/VMQ blends.

## 1. Introduction

With the incoming deorbit of the International Space Station, space agencies plan to leave the low Earth orbit in the hands of private industry and shift their focus towards Moon and Mars exploration [1,2]. This will likely foster the development of new extraplanetary missions and stimulate the demand for new solutions. Thus, the materials utilized in those missions need to prove to be reliable in extreme conditions. So far, all Mars rovers drive on wheels made of aluminum alloys. Metals, while resistant to Martian weather conditions, happen to be vulnerable to long-term dynamic fatigue, as shown in the reports of Curiosity wheel damage [3]. The material subjected to continuous deformation eventually failed and the wheel was damaged. This showed that a material resistant to fatigue, capable of providing elastic and damping properties is needed. This is especially important when considering future crewed missions that require material for dynamic sealing to secure the integrity of spacesuits and airlock doors. What is more, vibration reduction in fast-moving vehicles is crucial.

Properly designed rubber compounds emerge as excellent candidates to meet these requirements. Due to their inherent elasticity and fatigue resistance, they can provide the necessary performance under Martian conditions. Additionally, because Mars has a very low atmospheric pressure, such compounds must be free of volatile components like processing oils that could evaporate and contaminate the environment, simultaneously changing the properties of the rubber. As Mars’s atmosphere contains only trace levels of oxygen, the potential for oxidative degradation is greatly reduced [4], which will improve the long-term performance of rubber materials on Mars.

Rubbers, however, preserve their elastic properties only above a glass transition temperature (T_g_). T_g_ determines the temperature level below which segmental movement of rubber macromolecules stops, effectively turning a rubbery material into a brittle one. To withstand low temperatures on Mars, only rubbers with the lowest T_g_ can be used, like silicone rubber or butadiene rubber.

**Figure 1:**
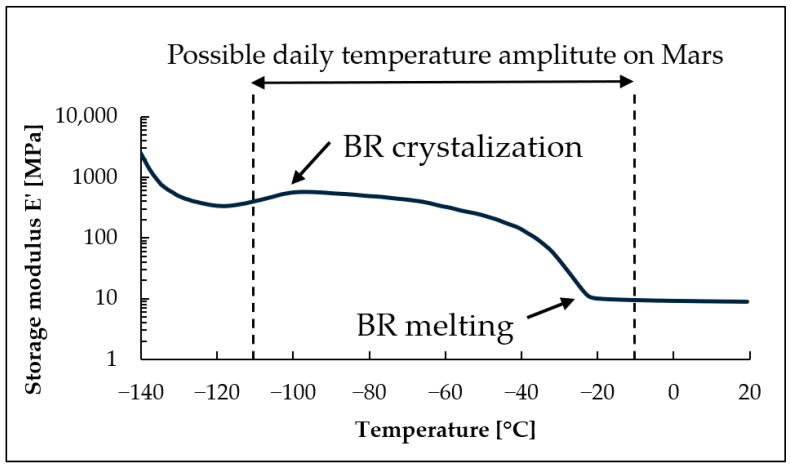
Storage modulus values for rubber material based on Buna CB24 BR (High-cis (>96%), neodymium-catalyzed butadiene rubber manufactured by Arlanxeo, The Netherlands) compared to the daily range of temperatures recorded on Mars by the Spirit rover [6]. Own DMA measurement performed on the DMA1 Dynamic Mechanical Analyzer, Mettler Toledo, USA, applying conditions described in Section 2.6.

Our prior research introduced the idea of blending butadiene rubber (BR) with vinyl methyl silicone rubber (VMQ), chosen for their low glass transition temperatures (T_g_ = −105 °C and T_g_ = −125 °C, respectively), to obtain a material capable of maintaining elastic properties in low temperatures. The compounds showed high radiation resistance and viable mechanical properties for various applications in Mars exploration missions [5]. The research focused on the low end of the temperature range, however, a different perspective allowed us to uncover an overlooked issue—since the daily temperature amplitude on Mars can reach 100 degrees, (e.g., from −10 °C to −110 °C) [6], the material on Mars will be subjected to a wide range of temperatures daily. The maximum temperature on Mars can reach 30 °C during the day on the equator and a minimum of −140 °C during the night in polar areas [7]. Rubber materials, throughout a range of temperatures, can undergo modifications in their structure, including glass transition and formation or the melting of crystalline phase. When such a temperature range is compared with changes in the storage modulus of a crystallizable, high-cis BR grade (Figure 1), it can be seen that a transition phase to a crystalline state lies within the daily Martian temperature range. This means that the material would undergo crystallization and melting cycles of polymer domains every day. While the crystallization can lead to reduced elasticity and increased permanent strain deformation [8], it also affects how much the material shrinks when the temperature decreases [9,10]. While the unstable volume of seals can be a potential threat to sealing properties, not much research can be found on how volume change affects interfacial leakage in rubber-sealed joints. Akulichev et al. suggested that for static joints, the low-temperature sealing properties can be improved by using a material with a matching thermal expansion coefficient to that of a seal housing material [11]. However, such a solution is very difficult to apply in practice.

In this research, BR/VMQ blends containing crystallizing and fully amorphous butadiene rubbers were compared to determine how the presence of a crystalline phase impacts the mechanical properties and shrinkage of the rubber compounds in low temperatures. Rubber compounds containing vinyl methyl silicone rubber, either semi-crystalline Buna CB24 BR or amorphous Buna CB550 BR, and sulfur-based vulcanization systems were compounded with either no filler, with silica, or carbon black. Oils are not present in the compound formulations to ensure no degassing would occur in the low-pressure Martian atmosphere. The operational temperature range of the blends is limited by the T_g_ of BR, which constitutes the continuous phase of the blends at low-temperature and BR thermal stability in the oxygen-free atmosphere of Mars. Vulcanization curves, static mechanical properties at low temperatures, dynamical mechanical properties, shrink at low temperatures, and differential scanning calorimetry were investigated.

## 2. Materials and Methods

### 2.1. Materials

Vinyl methyl silicone rubber (VMQ) containing one vinyl group per 99 methyl groups in the polymer backbone (Polimer^®^MV 1,0) was provided by Silikony Polskie, Nowa Sarzyna, Poland. High-cis neodymium butadiene rubber (BR), Buna^®^CB24, and medium-cis lithium BR, Buna^®^CB550, were provided by Arlanxeo, Geleen, The Netherlands. Rubber additives—activators: zinc oxide, stearic acid; accelerator: N-cyclohexyl-2-benzothiazole sulfenamide (CBS); curative: sulfur; antiozonant: N-(1,3-Dimethylbutyl)-N’-phenyl-p-phenylenediamine (6PPD) were supplied by Torimex Chemicals, Lodz, Poland. Carbon black (CB) N330 grade (BET surface area of 78 m^2^/g) was provided by Makrochem, Lublin, Poland. Precipitated silica Ultrasil^®^7000 (BET surface area of 170 m^2^/g) and bis(TriEthoxySilylPropyl)Disulfide (TESPD), Si266, were provided by Evonik Industries, Essen, Germany.

### 2.2. Elastomer Blends Preparation

Elastomer blends were prepared using laboratory mixer Plastograph^®^ Lab-Station (Brabender, Germany) by mixing butadiene rubber, silicone rubber, and remaining components as shown in the compound formulations presented in Table 1, following the two-step mixing procedure described in Table 2.

The utilization of a two-step mixing procedure involving high-temperature compounding was required for the in-situ silanization of silica. The samples filled with carbon black or without fillers were also prepared following this procedure. While it was confirmed that this may lead to a thermo-mechanical degradation of the polymer matrix and a slight deterioration of mechanical properties [12], such an approach was deemed necessary to obtain an objective comparison of the rubber compounds. The mixing process did not cause oxidative degradation, as can be seen in Figures S1 and S2 in the Supplementary Materials presenting Fourier transform infrared spectra of the pristine BRs and their blends with VMQ prepared at the elevated temperature.

### 2.3. Vulcanization Kinetics

Torque changes over time in the constant vulcanization temperature were investigated using the rubber process analyzer RPA2000 (Alpha Technologies, Hudson, OH, USA). From the torque curves, optimum cure time (t_90_, time to reach 90% of ΔM), scorch time (t_05_—time to reach 5% of ΔM), minimum torque (M_min_), maximum torque (M_max_), and torque increase (ΔM, M_max_ − M_min_) were obtained. The measurements were carried out at 160 °C for 60 min (30 min for samples with no filler). Additionally, the conventional cure rate index (CRI) of the rubber blends was calculated according to Equation (1), based on the work of Yehia and Stoll [13]. Also, the linear parts of the torque increases were analyzed by fitting linear functions with the R^2^ coefficient factor greater than 0.99.

(1) $$CRI=\frac{100}{t_{90}-t_{05}}$$

### 2.4. Vulcanization

Flat sheet and cylinder-shaped vulcanizates were prepared using steel molds and a home-made laboratory hydraulic press at a temperature of 160 °C and under 10 MPa of pressure. Vulcanization times of the compounds were based on the optimum cure times obtained from the vulcanization kinetics investigation (Table 3).

### 2.5. Static Mechanical Properties at Various Temperatures

Stress–strain curves were determined using Universal Testing Machine Z005 equipped with a thermostatically controlled chamber (ZwickRoell, Ulm, Germany). The temperature chamber was cooled with liquid nitrogen and the measurements were performed at −40 °C, −20 °C, 0 °C, and at room temperature. Tensile strength (TS), elongation at break (EB), moduli at 100%, 200%, and 300% of elongation (M100, M200, and M300, respectively), and stress at maximum elongation (T_Fmax_) were determined (T_Fmax_ was presented instead of TS for samples that did not break at maximum elongation of 1000%). The test was performed at a speed of 500 mm/min, utilizing type 2 dumbbell-shaped test pieces cut from flat-sheet vulcanizates, in accordance with the ISO 37 standard [14].

Vulcanized sheets were subjected to 25 thermal cycles consisting of submersion in liquid nitrogen for 10 s, followed by heating using a heat gun set to 50 °C, blowing from a distance of 15–20 cm until a room temperature was achieved. Afterwards, dumbbell-shaped specimens were cut from the sheets and tensile tests were performed.

### 2.6. Dynamic Mechanical Analysis

Values of storage modulus E’, loss modulus E’’, and tanδ were obtained for the temperature range from −145 °C to 30 °C using a DMA1 Dynamic Mechanical Analyzer (Mettler Toledo, Columbus, OH, USA). The heating/cooling rate was set to 2 K/min and the frequency was fixed at 10 Hz. Samples of rectangular cross-section (4 mm × 2 mm), obtained from the type 2 dumbbell-shaped test pieces (ISO 37 standard), were used for testing. Only the filled vulcanizates were successfully tested due to the low tear resistance of the unfilled vulcanizates, which were damaged during the DMA investigation.

### 2.7. Thermal Shrinkage Measurement

The displacement of the compression clamps (representing the height change of the cylindrical-shaped specimen) was measured during cooling (from 0 °C to −140 °C at 5 K/min rate) and heating cycles (from −140 °C to 0 °C at 5 K/min rate). The measurement was performed using the Dynamic Mechanical Analyzer DMA Q800 (TA Instruments, New Castle, DE, USA) in a compression static mode. The tests were performed on cylindrical samples of 15 mm in diameter and 8 mm in height by measuring the linear changes in the samples’ height.

### 2.8. Differential Scanning Calorimetry

Thermal analysis using differential scanning calorimetry (DSC) was performed using a DSC1 calorimeter (Mettler Toledo, Greifensee, Switzerland) calibrated based on standards (indium, n-octane). The measurement procedure was as follows: segment I: cooling from 25 °C to −150 °C, segment II: heating from −150 °C to 25 °C, segment III: cooling from 25 °C to −150 °C, segment IV: heating from −150 °C to 25 °C. Heat flow was recorded for the second heating. The heating/cooling rates were set to 5 K/min. Liquid nitrogen was used as a cooling agent and the nitrogen (flow rate 80 mL/min) was applied as the protective gas. The mass of the samples was in the range of 15–20 mg.

## 3. Results and Discussion

### 3.1. Vulcanization Kinetics

**Figure 2:**
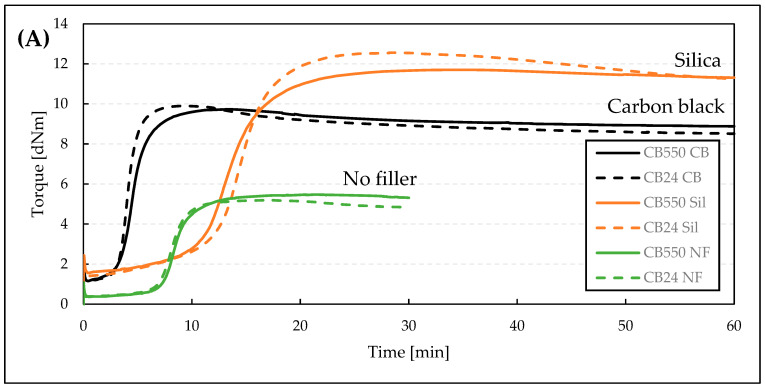

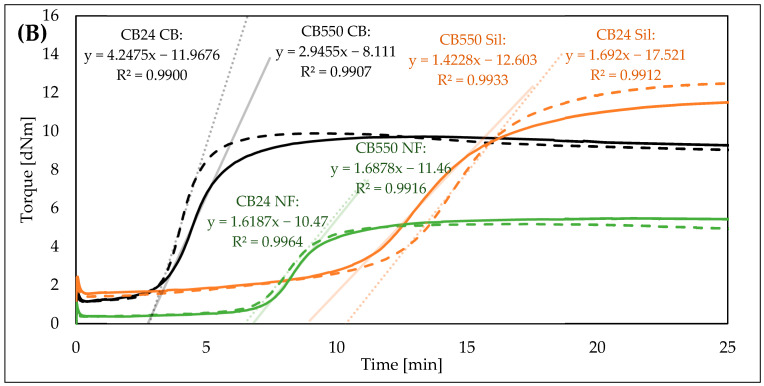
(**A**)**:** Vulcanization curves of BR/VMQ blends containing either CB24 or CB550 butadiene rubber and different fillers; (**B**): Vulcanization curves with slope equations for the linear segments of the curing process.

Vulcanization kinetics parameters are shown in Table 3 and the full curves are shown in Figure 2.

A slight increase in M_max_ can be observed for CB24 samples with fillers (CB24 CB and CB24 Sil) (Table 3) and the slopes of their cure curves are steeper, suggesting faster vulcanization (Figure 2). This is also represented in the CRI values where the curing rates are clearly higher for CB24 samples, except for silica-filled samples where the CRI is very similar for both types of BR. However, the torque increase is clearly faster for the CB24 at the start of vulcanization. This is due to a flocculation effect occurring before the vulcanization starts and is visible as a linear increase of torque up to 10 min of the test. As a result, the t_05_ parameter values of the silica-filled compounds are affected and instead of showing the beginning of vulcanization, they show the progress of flocculation. This, in turn, affects the CRI measurement (Equation (1)). To supplement this, Figure 2B shows the slopes of the linear segments of the curing curves. Through both visual analysis and obtained slope coefficients, the results are in agreement with the findings of Marzocca et al. who showed that the butadiene rubber rate of curing (measured as the slope of the torque curve during the curing process) is higher for rubbers with higher cis content [15]. Also, this is in line with the literature findings, which show that the addition rate of sulfur to the BR chain increases with the increasing content of 1,4-mer configuration [16]. The addition of carbon black noticeably increased the curing rate due to its catalytic effect on sulfur vulcanization [17], while the addition of silica increased the curing time as a result of curative adsorption on the silica surface [18,19,20]. Both of those effects confirm our previous research results [12], showing that the addition of N330 grade carbon black into BR/VMQ blends speeds up vulcanization, while the incorporation of precipitated silica (Ultrasil^®^7000) slows it down.

**Table 3:** Scorch time (t_05_), optimum cure time (t_90_), minimum torque (M_min_), maximum torque (M_max_), torque increase (ΔM), and the cure rate index (CRI) of the BR/VMQ compounds.

Sample Denotation	t_05_ [min]	t_90_ [min]	M_max_ [dNm]	M_min_ [dNm]	ΔM [dNm]	CRI [%/min]
CB550 CB	2.6	6.9	9.7	1.2	8.6	23.42
CB24 CB	2.8	5.5	9.9	1.2	8.8	37.04
CB550 Sil	6.8	18.8	11.7	1.6	10.1	8.35
CB24 Sil	6.4	18.7	12.6	1.4	11.2	8.15
CB550 NF	6.2	11.5	5.5	0.4	5.1	18.83
CB24 NF	5.5	10.1	5.2	0.4	4.8	21.65

### 3.2. Static Mechanical Properties at Various Temperatures

**Figure 3:**
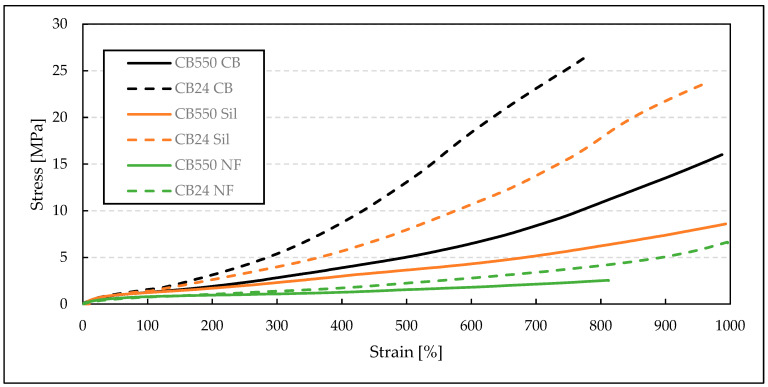
Stress–strain curves of BR/VMQ blends containing either CB24 or CB550 butadiene rubber and different fillers, measured at −40 °C.

**Figure 4:**
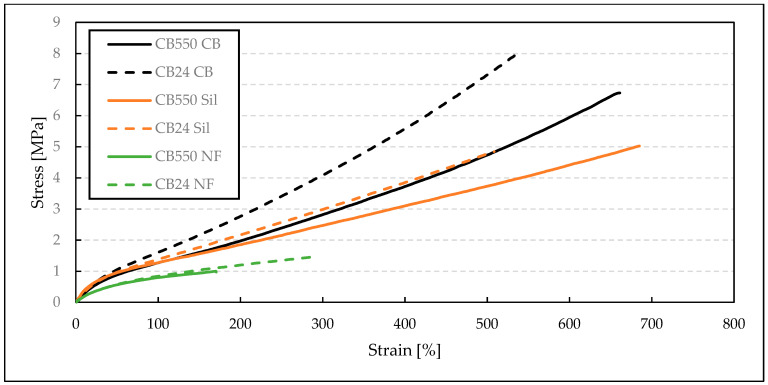
Stress–strain curves of BR/VMQ blends containing either CB24 or CB550 butadiene rubber and different fillers, measured at room temperature.

For tensile strength measurements (Table 4), it is clearly seen that the blends with semi-crystalline CB24 butadiene, containing mainly cis-mers, have shown greater TS values when compared to their amorphous counterparts. This improvement in mechanical properties is visible below the crystallization temperature, where the crystalline structure increases the moduli and tensile strength of the BR/VMQ blends. For the filled samples, the blends containing CB24 also showed lower EB. Both EB and TS increased with decreasing temperatures, which is evident when comparing results at −40 °C (Figure 3) and at room temperature (Figure 4). This is caused by the Gough–Joule effect, which describes the contraction of rubber molecules to entropically favorable combs after strain-induced elongation [21]. This results in a higher stress concentration with an increasing temperature effectively reducing the EB value. Therefore, at lower temperatures, the TS and EB parameters for the BR/VMQ blends are higher, as the energy of the system is lower and polymer chains have less energy to contract the strain-induced alignment, effectively reducing the amount of stress in the material. Lastly, the improvement in static mechanical properties caused by fillers is clearly visible. Both silica and carbon black increase the tensile strength of the material, with the latter having a greater reinforcing effect regardless of temperature or the type of BR. This is in line with our previous results, which show that CB improves the mechanical properties of BR/VMQ blends more effectively than the silica/silane system due to more even interactions with both rubbers [12]. This is due to the silane in the silica/silane system coupling silica mostly to the BR phase due to its higher level of unsaturation [22].

**Figure 5:**
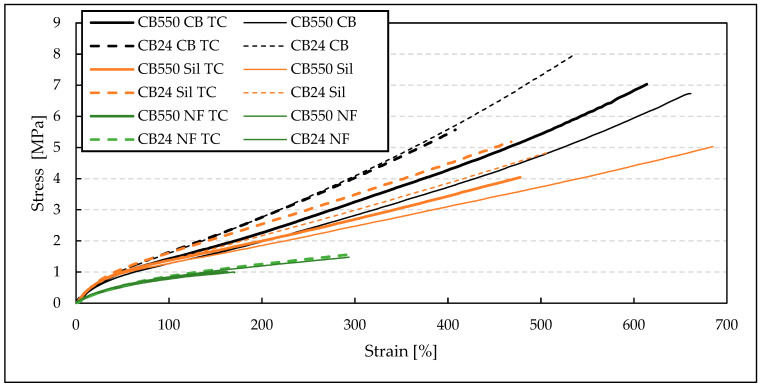
Stress–strain curves of BR/VMQ blends before and after 25 thermal cycles (TC) containing either CB24 or CB550 butadiene rubber and different fillers, measured at room temperature.

The results of mechanical properties testing of the blends before and after 25 thermal cycles (TC) are shown in Figure 5 and Table 5.

The mechanical properties of the unfilled blends show little to no change after the TC. On the contrary, the filled blends exhibit a visible change in their mechanical properties. This suggests a morphological rearrangement of macromolecules adsorbed on the surface of the fillers. Thermal expansion of rubber matrix differs from filler particles, which leads to stress generation in the polymer/filler interphase during TC. As a result, the mechanical properties of the BR/VMQ blends change noticeably. In general, a decrease in EB is observed for all blends after TC, while the value TS changes without correlation to BR type of filler type. Crystallization of BR seems not to affect the mechanical properties of the blends after TC.

### 3.3. Dynamic Mechanical Analysis

**Figure 6:**
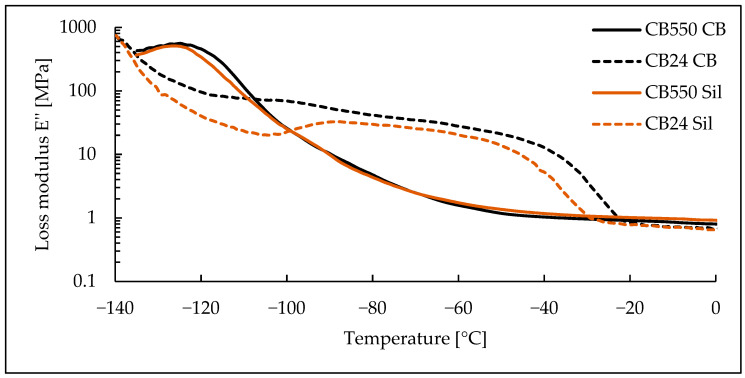
Loss modulus measurements for BR/VMQ blends containing either CB24 or CB550 butadiene rubber and carbon black (CB) or silica (Sil) filler.

**Figure 7:**
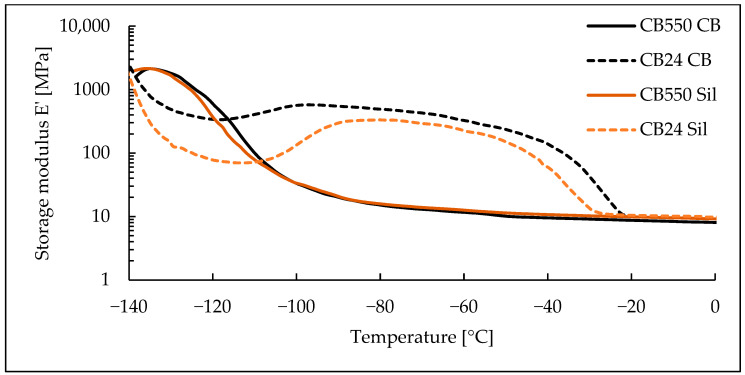
Storage modulus measurements for BR/VMQ blends containing either CB24 or CB550 butadiene rubber and carbon black (CB) or silica (Sil) filler.

**Figure 8:**
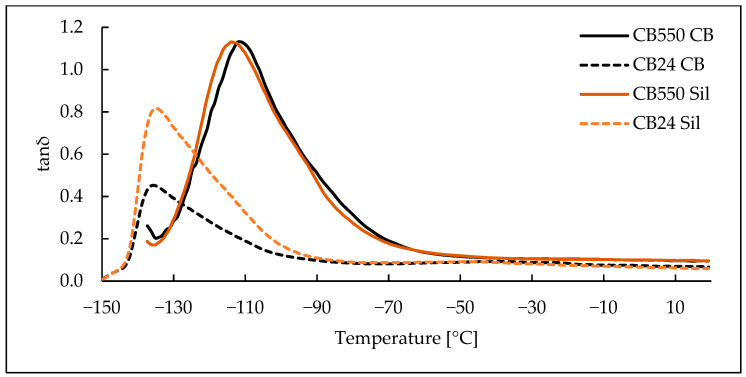
Tanδ measurements for BR/VMQ blends containing either CB24 or CB550 butadiene rubber and carbon black (CB) or silica (Sil) filler.

Based on the DMA results, shown as loss and storage moduli in function of temperature (Figure 6 and Figure 7), the lack of crystalline phase in CB550 is clearly visible. When compared to the temperature range shown in Figure 1, samples containing CB550 show a plateau of moduli values, indicating relatively stable dynamic mechanical properties throughout most of the daily temperature range of Mars. However, the loss modulus value changes more with decreasing temperature than storage modulus, indicating more energy dissipation by the compounds at lower temperatures. For tanδ curves shown in Figure 8, the peak is shifted towards higher temperatures, indicating higher T_g_ of the amorphous BR. The difference in T_g_ between the compared butadiene rubbers is expected, as CB550 has a higher content of trans-1,4 and vinyl-1,2 mers (52–53% and 9–10%, respectively) than CB24, in which their content is negligible. The shift to higher T_g_ with increased vinyl content is in line with the literature findings showing that T_g_ of butadiene rubber is strongly dependent on the microstructure of the polymer and increases proportionally to vinyl-1,2 content [23]. The peak value is also higher, indicating more damping properties of the material.

### 3.4. Thermal Shrinkage Measurement

**Figure 9:**
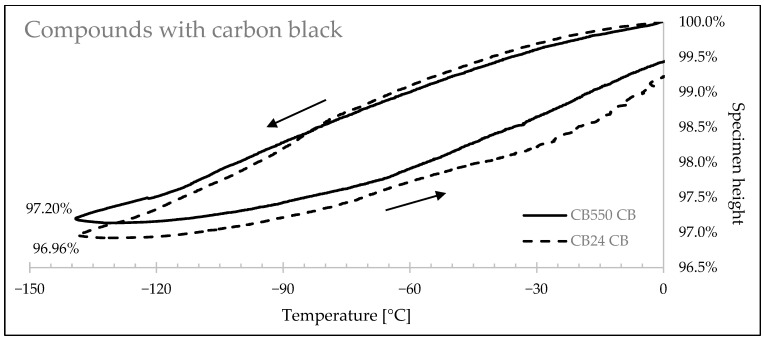
Rubber specimen height changes with the change of temperature for samples with carbon black filler.

**Figure 10:**
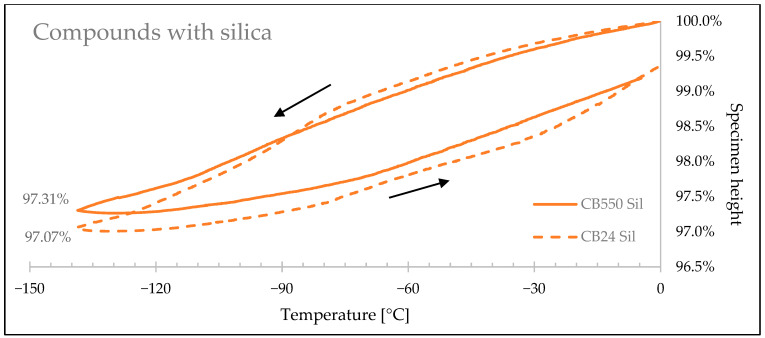
Rubber specimen height changes with the change of temperature for samples with silica filler.

**Figure 11:**
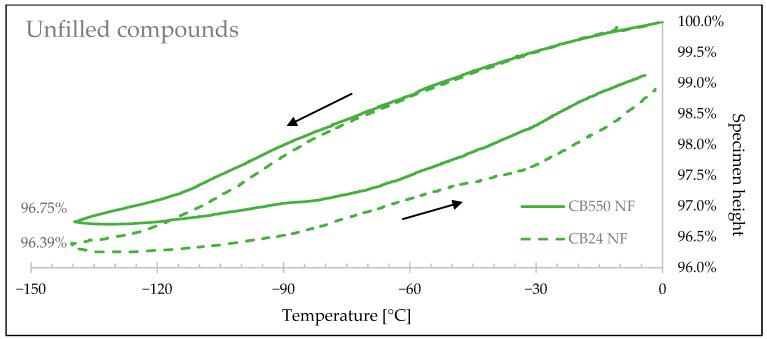
Rubber specimen height changes with the change of temperature for samples with no fillers.

Figure 9, Figure 10 and Figure 11 show how the rubbers’ linear sizes change during the cooling/heating cycles. Faster shrinkage of the compounds containing crystallizing CB24 butadiene can be observed below −85 °C caused by the formation of the crystalline phase of the macromolecules occupying smaller space volume. Table 6 presents a relative decrease in shrinkage, showing that the use of amorphous butadiene rubber in the BR/VMQ blend reduced material shrink by 6.71% for samples with carbon black, by 8.36% for samples with silica, and by 11.63% for unfilled samples.

The relative reduction of the shrink is significant when an amorphous grade BR is used. However, the total difference is very small, oscillating between 0.24% and 0.36% of the samples’ height. These differences can be easily compensated in practice by designing thicker seals or applying a more flexible cross-section shape. Only in cases of applications requiring very thin sealing gaskets may a leaking problem occur.

### 3.5. Differential Scanning Calorimetry

**Figure 12:**
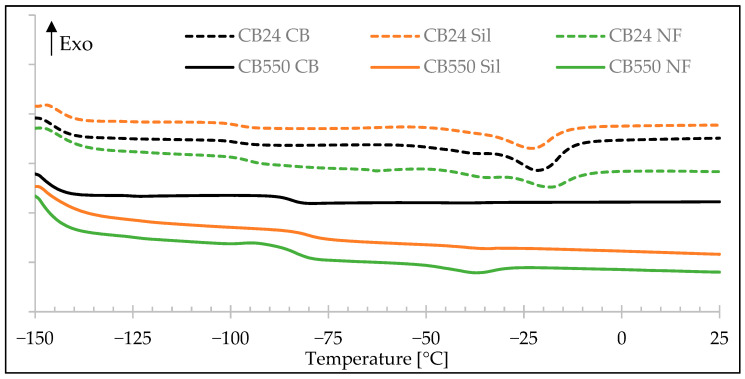
Second heating heat flow curves for all BR/VMQ blends. Dotted lines represent blends with semi-crystalline CB24 butadiene rubber, solid lines represent blends with amorphous CB550 butadiene rubber.

The DSC results (Figure 12, Table 7) show the lack of BR phase crystallization peak for CB550 samples, which is present for the CB24 at around −18 °C to −24 °C. VMQ crystallization peak can be observed at around −35 °C to −40 °C. Its disappearance from the filled blends can be explained by the adsorption of silicone rubber on filler particles which immobilizes rubber chains preventing them from forming crystalline structures [22,24]. This effect is more pronounced for the compounds filled with silica than the compounds containing carbon black, most likely due to stronger polar interactions between the free electron pairs of oxygen atoms in VMQ chains and silanols present on the silica surface [25]. The glass transition of the butadiene phase is higher when CB550 is used, this was also observed in the DMA measurement and is attributed to the composition of the rubber chains with a higher content of vinyl-1,2 mers.

The ΔH_m_ value of VMQ rubber containing 1% of vinyl groups is not present in the literature because of the very specific vinyl content; therefore, our results will be compared to the properties of polydimethylsiloxane (PDMS), keeping in mind that the presence of vinyl groups in VMQ can act as small branches decreasing the ability of close packing of the polymer chains, therefore reducing the crystallinity and the ΔH_m_ value. The most common value for the enthalpy of melting of uncured PDMS is around 35 J/g [26,27], which is more than twice of what was recorded in this study (12.53 J/g for CB24 CB, 14.51 J/g for CB24 Sil, and 17.48 J/g for CB24 NF). Interestingly, VMQ forms a higher amount of the crystalline phase when the semi-crystalline CB24 is used for blending. This might be a result of the nucleating effect of BR crystals—BR crystallizes at higher temperatures than VMQ and its crystallites can serve the latter as nucleating sites. Alternatively, CB24 adsorption on the surface of the fillers may be greater than CB550, resulting in a lower area for VMQ adsorption and immobilization.

In the case of BR, uncured high-cis BR is reported to have its melting enthalpy around 46 J/g [8,28] and the values obtained in this study are 9.04 J/g for the unfilled, and 20.19 J/g and 25.05 J/g for the samples with silica and carbon black, respectively. This is again a value lower than that of the literature reference. This is not an uncommon effect considering the influence of cross-linking on polymer crystallization. Poor S. M. showed in his doctoral dissertation that the ΔH_m_ of high-cis BR decreases by about half for cured samples [29], which is in line with the results obtained in this study for CB24 samples with fillers. In contrast to VMQ, the addition of the fillers to the BR/VMQ blends results in an increase of the BR crystalline phase amount (ΔH_m(BR)_). This can be explained by the nucleating effect of the fillers’ particles, in which surface interactions with BR macromolecules trigger the formation of crystallites.

The decrease in melting enthalpy of both VMQ and BR caused by vulcanization represents the decreased mobility of cross-linked polymer chains and therefore their reduced ability to rearrange into packed crystalline structures.

## 4. Conclusions

Rubber blends containing vinyl methyl silicone rubber (VMQ) and two types of butadiene rubber (BR), semi-crystalline high-cis CB24 and amorphous CB550, were prepared and their properties were compared. Additionally, the effect of filler (carbon black, silica, or no filler) was investigated. The use of amorphous butadiene rubber allowed for a decrease in the material’s shrinkage in extremely low temperatures (shrinkage decrease of 6.71% for samples with carbon black, 8.36% for samples with silica, and 11.63% for samples with no filler). The lack of transition to the crystalline phase allowed the material to exhibit more consistent damping and elastic dynamic properties throughout the temperature range equivalent to the daily temperature amplitude on Mars. CB550 also exhibited higher glass transition temperatures due to a higher vinyl-1,2 mers content and lower tensile strength at different temperatures because of the lack of a reinforcing crystalline phase.

The formation of the BR crystalline phase greatly improved the mechanical properties of the BR/VMQ blends, showing a significant reinforcing potential.

Due to the limited research on sealing shrink in sub-zero temperatures and its impact on sealing properties, it cannot be conclusively assessed whether the use of amorphous CB550 is justified. Additionally, material behavior over multiple cycles must be assessed, especially for crystallizing rubber, as stress and microcracking may accumulate over time.

Further investigations into the material’s sealing properties and cyclic thermal testing are planned to be carried out in the future.

The presented results are aimed at broadening knowledge about the behavior of BR/VMQ rubber blends for Mars applications. Although the development of rubber for Mars is still in progress, based on the current results, we firmly believe that we are on a prospective path towards achieving this goal.

## Figures and Tables

**Table 1:** Formulations of the BR/VMQ compounds. Amounts are given in parts per hundred parts of rubber by weight (phr). CB, Sil, and NF suffixes denote the filler—carbon black, silica, or an unfilled sample, respectively.

Samples Denotation Component [phr]	CB24 CB	CB550 CB	CB24 Sil	CB550 Sil	CB24 NF	CB550 NF
BR CB24 (semi-crystalline)	80	-	80	-	80	-
BR CB550 (amorphous)	-	80	-	80	-	80
VMQ polymer MV 1,0	20	20	20	20	20	20
Carbon Black N330	30	30	-	-	-	-
Silica Ultrasil 7000	-	-	30	30	-	-
Si 266 (TESPD)	-	-	3	3	-	-

Other ingredients [phr]: Vulcanization activators: Zinc oxide—3; Stearic acid—3. Vulcanization accelerator: N-cyclohexyl-2-benzothiazole sulfenamide (CBS)—1.6. Vulcanization agent: Sulfur—1.2. Antiozonant: N-(1,3-Dimethylbutyl)-N’-phenyl-p-phenylenediamine (6PPD)—2.

**Table 2:** Two-step mixing procedure of the BR/VMQ compounds.

Time [min]	Action	Rotor Speed [rpm]	Temperature [°C]
Step 1			
0:00	Add BR and VMQ	20	Room temp.
1:00	Increase rotor speed	60	Room temp.
6:00	Add filler, silane (if applicable), ZnO, stearic acid, 6PPD	60	100
7:00	Increase rotor speed, reach 130 °C	80–90	100
12:30	Decrease rotor speed	60	130
15:30	Stop mixing	0	130
Step 2			
0:00	Add pre-mix	30	50
1:00	Increase rotor speed	60	50
3:00	Add CBS, sulfur	60	70
5:00	Stop mixing	0	70

**Table 4:** Tensile strength (TS) or stress at maximum elongation T_Fmax_ (marked in red color), elongation at break (EB), and moduli at 100%, 200%, and 300% of elongation (M100, M200, and M300) of all BR/VMQ blends, measured at room temperature, 0 °C, −20 °C, and −40 °C.

Samples Denotation	Temperature [°C]	M100 [MPa]	M200 [MPa]	M300 [MPa]	TS or T_Fmax_ [MPa]	EB [%]
CB24 CB	Room Temp.	1.6 ± 0.1	2.8 ± 0.1	4.1 ± 0.2	7.5 ± 0.7	520 ± 49
	0	1.6 ± 0.03	2.8 ± 0.1	4.3 ± 0.1	11.1 ± 1.6	649 ± 58
	−20	1.6 ± 0.1	2.9 ± 0.1	4.6 ± 0.1	11.6 ± 0.7	552 ± 18
	−40	1.6 ± 0.1	3.3 ± 0.2	5.7 ± 0.3	25.6 ± 1.2	742 ± 33
CB550 CB	Room Temp.	1.3 ± 0.04	2.0 ± 0.1	2.9 ± 0.1	6.8 ± 0.8	654 ± 55
	0	1.2 ± 0.01	1.95 ± 0.01	2.85 ± 0.01	10.7 ± 0.6	821 ± 25
	−20	1.2 ± 0.03	1.9 ± 0.1	2.9 ± 0.1	13.7 ± 1.8	897 ± 64
	−40	1.3 ± 0.04	1.94 ± 0.04	2.87 ± 0.03	14.3 ± 2.8	>1000
CB24 Sil	Room Temp.	1.5 ± 0.05	2.3 ± 0.1	3.1 ± 0.1	4.8 ± 0.9	489 ± 97
	0	1.6 ± 0.03	2.5 ± 0.1	3.6 ± 0.1	7.8 ± 1.2	619 ± 76
	−20	1.4 ± 0.1	2.5 ± 0.1	3.6 ± 0.1	18.2 ± 0.9	960 ± 28
	−40	1.4 ± 0.02	2.68 ± 0.04	4.06 ± 0.04	23.0 ± 1.1	943 ± 26
CB550 Sil	Room Temp.	1.3 ± 0.03	1.84 ± 0.04	2.46 ± 0.04	5.1 ± 0.8	686 ± 114
	0	1.2 ± 0.03	1.80 ± 0.03	2.41 ± 0.04	7.7 ± 0.2	952 ± 32
	−20	1.2 ± 0.04	1.73 ± 0.07	2.34 ± 0.09	8.2 ± 0.4	>1000
	−40	1.2 ± 0.03	1.70 ± 0.02	2.33 ± 0.02	8.6 ± 0.2	>1000
CB24 NF	Room Temp.	0.8 ± 0.04	1.16 ± 0.03	1.46 ± 0.04	1.4 ± 0.2	291 ± 49
	0	0.8 ± 0.01	1.15 ± 0.02	1.43 ± 0.03	2.7 ± 0.4	677 ± 108
	−20	0.8 ± 0.03	1.08 ± 0.02	1.36 ± 0.02	4.3 ± 0.3	954 ± 22
	−40	0.8 ± 0.01	1.07 ± 0.01	1.31 ± 0.01	6.6 ± 0.1	>1000
CB550 NF	Room Temp.	0.8 ± 0.02	-	-	1.0 ± 0.1	181 ± 35
	0	0.8 ± 0.01	0.99 ± 0.01	-	1.2 ± 0.1	282 ± 30
	−20	0.7 ± 0.01	0.95 ± 0.03	1.14 ± 0.05	1.23 ± 0.01	348 ± 13
	−40	0.8 ± 0.03	0.95 ± 0.03	1.10 ± 0.03	2.5 ± 0.3	803 ± 64

**Table 5:** Tensile strength (TS) and elongation at break (EB) of all BR/VMQ blends before and after 25 thermal cycles (TC), measured at room temperature.

Samples Denotation	CB24 CB	CB550 CB	CB24 Sil	CB550 Sil	CB24 NF	CB550 NF
TS before TC [MPa]	7.5 ± 0.7	6.8 ± 0.8	4.8 ± 0.9	5.1 ± 0.8	1.4 ± 0.2	1.0 ± 0.1
TS after TC [MPa]	5.6 ± 0.2	7.0 ± 1.0	5.2 ± 0.2	4.2 ± 0.4	1.5 ± 0.2	1.0 ± < 0.1
EB before TC [%]	520 ± 49	654 ± 55	489 ± 97	686 ± 114	291 ± 49	181 ± 35
EB after TC [%]	407 ± 15	613 ± 53	469 ± 37	484 ± 72	271 ± 50	166 ± 10

**Table 6:** Height and volume changes were recorded upon reaching −140 °C for all tested BR/VMQ compounds. For each paired result of CB24 and CB550, relative changes are presented in the form of shrink difference. Volume changes were calculated under the assumption that the shrink is isotropic.

Sample Denotation	Height Change at −140 °C	Linear Shrink Difference	Volume Change at −140 °C	Volumetric Shrink Difference
CB24 CB	3.08%	6.91%	8.95%	6.71%
CB550 CB	2.86%		8.35%	
CB24 Sil	2.99%	8.59%	8.71%	8.36%
CB550 Sil	2.73%		7.98%	
CB24 NF	3.75%	12.03%	10.82%	11.63%
CB550 NF	3.30%		9.56%	

**Table 7:** Glass transition temperatures (Tg_(VMQ)_, Tg_(BR)_), melting temperatures (T_m(VMQ)_, T_m(BR)_), and melting enthalpy values per unit of rubber mass in the sample (ΔH_m(VMQ)_, ΔH_m(BR)_) obtained from DSC measurements during second heating.

Samples Denotation	Tg_(VMQ)_ [°C]	T_m(VMQ)_ [°C]	ΔH_m(VMQ)_ [J/g_VMQ_]	Tg_(BR)_ [°C]	T_m(BR)_ [°C]	ΔH_m(BR)_ [J/g_BR_]
CB550 CB	−125.2	−40.4	0.78	−84.8	n/a	
CB24 CB	−126.6	−36.1	12.53	−98.0	−21.5	25.05
CB550 Sil	−125.4	−36.9	1.91	−81.0	n/a	
CB24 Sil	−125.5	n/a		−98.1	−23.2	20.19
CB550 NF	−124.5	−37.9	14.51	−85.5	n/a	
CB24 NF	−124.5	−34.8	17.48	−96.2	−18.6	9.04

## Data Availability

The original data presented in the study are openly available in the ResearchGate profile of the first author at [DOI: 10.13140/RG.2.2.10719.47520].

## Supplementary Materials

The following supporting information can be downloaded at: https://www.mdpi.com/article/10.3390/ma17194857/s1, FTIR data comparing pristine butadiene rubbers and their respective BR/VMQ blends after mixing according to the procedure described in Section 2.2 is available in Supplementary Materials. Figure S1. FTIR spectra of the pristine CB550 butadiene rubber (BR) and the CB550 BR blended with Vinyl Methyl Silicone Rubber (VMQ) containing one vinyl group per 99 methyl groups in the polymer backbone. The blending process was done according to the procedure described in Section 2.2. Figure S2. FTIR spectra of the pristine CB24 butadiene rubber (BR) and the CB24 BR blended with Vinyl Methyl Silicone Rubber (VMQ) containing one vinyl group per 99 methyl groups in the polymer backbone. The blending process was done according to the procedure described in Section 2.2.
