# Supplementary material for: Effect of Butadiene Rubber Crystallization on Low-Temperature Properties of Butadiene/Silicone Rubber Blends with Potential for Mars Applications
Source: Materials (Basel). 2024 Oct 2;17(19):4857. doi: 10.3390/ma17194857 (PMC11478136; doi:10.3390/ma17194857)
Supplement: Supplementary file 1 [file materials-17-04857-s001.zip › materials-3202922-supplementary.pdf]

Supplementary Materials

to the article *Effect of Butadiene Rubber Crystallization on Low-Temperature Properties of Butadiene/Silicone Rubber Blends for Mars Applications*.

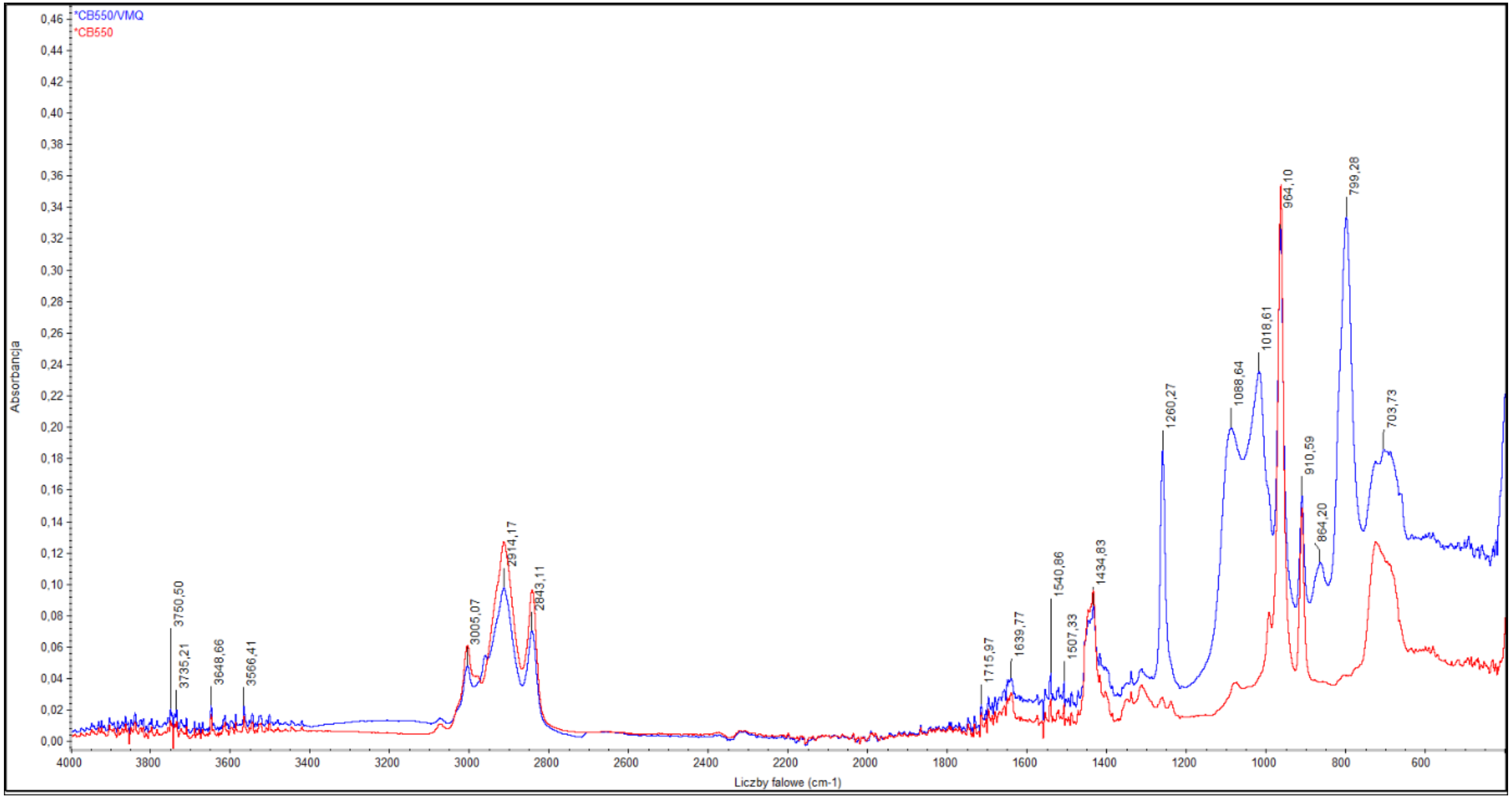

Figure S1. FTIR spectra of the pristine CB550 butadiene rubber (BR) and the CB550 BR blended with Vinyl Methyl Silicone Rubber (VMQ) containing one vinyl group per 99 methyl groups in the polymer backbone. The blending process was done according to the procedure described in section 2.2.

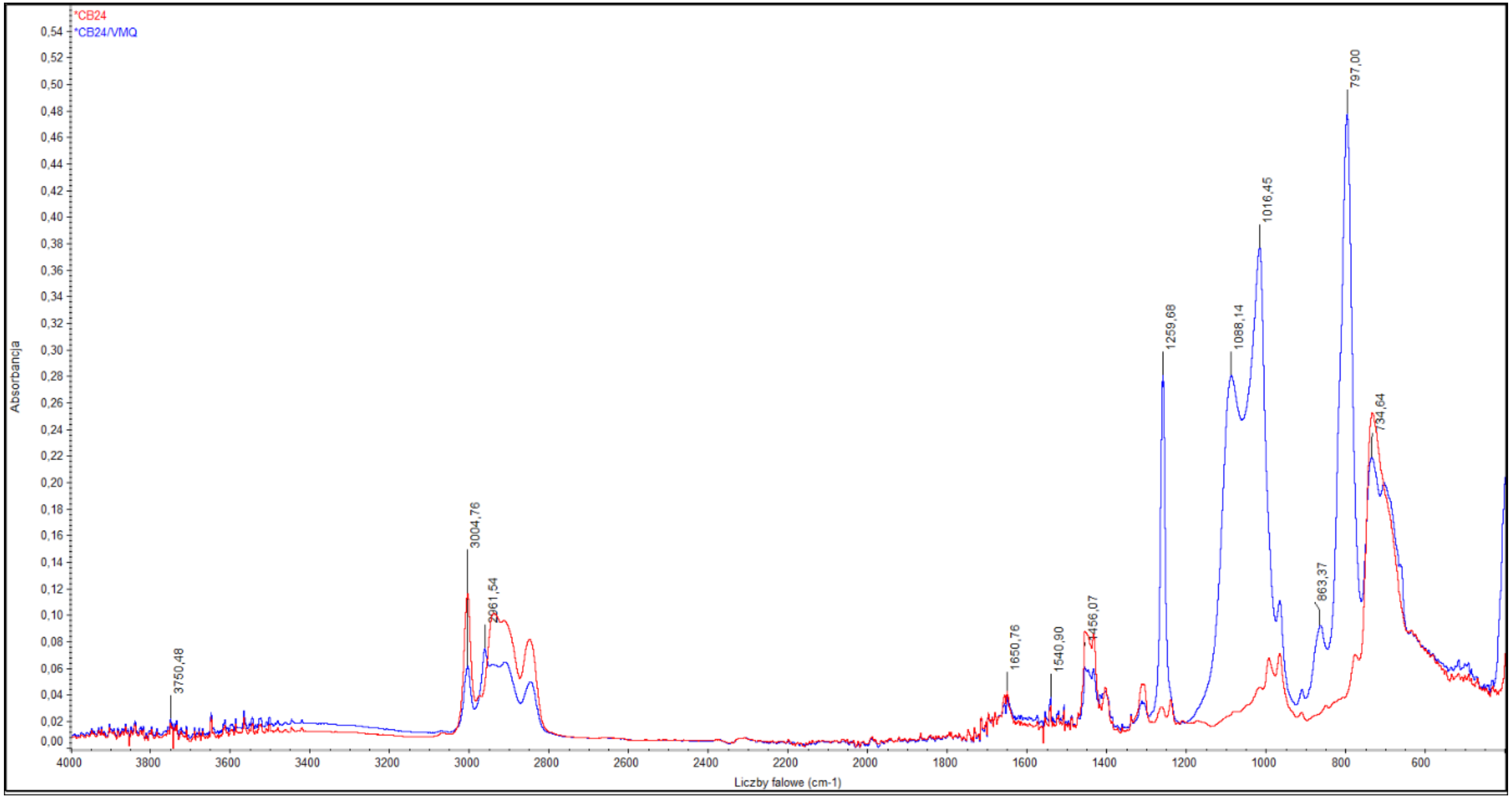

Figure S2. FTIR spectra of the pristine CB24 butadiene rubber (BR) and the CB24 BR blended with Vinyl Methyl Silicone Rubber (VMQ) containing one vinyl group per 99 methyl groups in the polymer backbone. The blending process was done according to the procedure described in section 2.2.
